# Preanalytical mystery: falsely elevated intact parathyroid hormone due to sampling from a grafted forearm

**DOI:** 10.11613/BM.2025.031002

**Published:** 2025-10-15

**Authors:** Yu-Wei Tseng, Chun-Chieh Yeh, Che-Yi Chou, Si-Yu Chen, Tze-Kiong Er

**Affiliations:** 1 Division of Laboratory Medicine, Asia University Hospital, Asia University, Taichung, Taiwan; 2 Department of Surgery, Asia University Hospital, Taichung, Taiwan; 3 Division of Nephrology, Asia University Hospital, Taichung, Taiwan; 4 Department of Medical Laboratory Science and Biotechnology, Asia University, Taichung, Taiwan

**Keywords:** case report, preanalytical error, iPTH, sampling site, parathyroid autotransplantation

## Abstract

Spurious elevations in intact parathyroid hormone (iPTH) can lead to unnecessary interventions. We describe a dialysis patient who developed unexpectedly high iPTH concentrations months after total parathyroidectomy with forearm autotransplantation (TPTX-AT). Blood samples had been collected from the grafted forearm, resulting in falsely elevated iPTH values. Once the sample was collected from the non-grafted arm, iPTH concentrations normalized. This case highlights the importance of sampling site awareness in laboratory diagnostics and demonstrates how implementing a simple laboratory information system (LIS)-based protocol can prevent misinterpretation.

## Graphical abstract

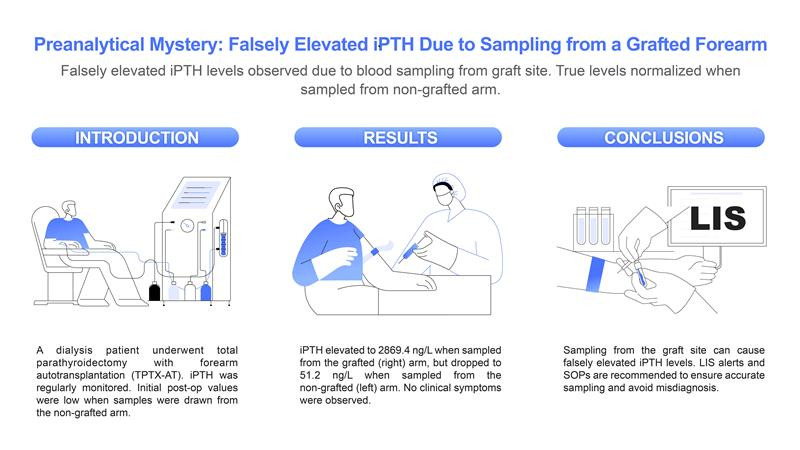

## Highlights

• Falsely elevated intact parathyroid hormone (iPTH) concentrations due to sampling from autotransplanted parathyroid site

• Misleading iPTH results raising concern for recurrent hyperparathyroidism

• Normalized iPTH upon contralateral arm sampling confirming sampling-site artifact

• Implementation of LIS-based alerts and staff training for proper sampling

• Emphasizes the importance of long-term documentation of sampling sites after parathyroidectomy

## Introduction

Secondary hyperparathyroidism (SHPT) is a frequent complication among patients with end-stage renal disease (ESRD). Secondary hyperparathyroidism is marked by excessive production and release of parathyroid hormone (PTH), accompanied by progressive enlargement and hyperplasia of the parathyroid glands (*1*). In some cases, elevated parathyroid hormone concentrations may persist even after successful kidney transplantation, despite normalization of standard biochemical markers (*2*). Nowadays, total parathyroidectomy with forearm autotransplantation (TPTX-AT) is a well-established treatment for secondary hyperparathyroidism (*3*).

Monitoring PTH concentrations is essential in managing patients with ESRD, particularly those undergoing chronic hemodialysis.

Previously, Khalil *et al.* reported that blood samples collected from the forearm with parathyroid autografts can yield spuriously elevated PTH concentrations due to localized hormone secretion. Although this preanalytical artifact is recognized in the immediate postoperative period, its potential to mislead results during long-term follow-up is often underappreciated (*4*). Consistent with these observations, Başcı *et al.* also described falsely elevated intact parathyroid hormone (iPTH) concentrations when blood was drawn from a forearm graft following total parathyroidectomy with TPTX-AT (*5*). We present a case demonstrating this issue and the quality improvement measures implemented. This case is aligned with Biochemia Medica’s emphasis on uncovering practical preanalytical challenges and educating laboratory professionals (*6*).

## Laboratory analyses

This case expands upon prior findings by demonstrating that sampling-site errors can have a delayed yet clinically significant impact on patient management in the dialysis setting. A 61-year-old male patient with ESRD on regular hemodialysis underwent TPTX-AT in October 2024, with autograft placement in his right forearm. Postoperatively, iPTH concentrations drawn from the left (non-grafted) arm declined sharply from 1037.0 ng/L preoperatively to 9.2 ng/L within 7 days. The surgical outcome was initially interpreted as successful. Blood samples were collected using serum separator tubes (SST) and processed within 2 hours of collection. Intact PTH concentrations were measured using a two-site immunoenzymatic (“sandwich”) chemiluminescent immunoassay (Beckman Coulter Access, UniCel DxI 800 platform, Brea, USA), with an analytical sensitivity of 1 ng/L.

During routine iPTH monitoring in February 2025, a blood sample was inadvertently drawn from the right forearm, where the parathyroid graft was located, yielding an iPTH concentration of 2869.4 ng/L and raising concern for recurrent hyperparathyroidism. A repeated test on March 14, also collected from the grafted arm, showed a persistently elevated iPTH concentration of 1936.0 ng/L. Despite these markedly elevated values, the patient’s serum calcium was 1.95 mmol/L (reference range: 2.15-2.58 mmol/L), and phosphorus was 1.84 mmol/L (reference range: 0.81-1.61 mmol/L), with no clinical signs or symptoms suggestive of recurrence. In April, a sample obtained from the non-grafted left arm revealed a normalized iPTH concentration of 51.2 ng/L. Table 1 demonstrates the chronological fluctuation of iPTH values in relation to blood sampling sites, highlighting the impact of graft-site collection.

**Table 1:** Intact parathyroid hormone (iPTH) concentrations and sampling sites in the patient over time

**Date**	**iPTH (ng/L)**	**Arm (Site)**
2024-01-03	1037.2	Left (AVF)
2024-04-03	1037.0	Left (AVF)
2024-06-07	1989.4	Left (AVF)
2024-07-04	1879.1	Left (AVF)
2024-09-02	1925.2	Left (AVF)
2024-10-03	1772.7	Left (AVF)
2024-11-01	13.4	Left (AVF)
2024-11-06	9.2	Left (AVF)
2025-01-01	55.8	Left (AVF)
2025-02-25	2869.4	Right (Graft)
2025-03-14	1936.0	Right (Graft)
2025-04-02	51.2	Left (AVF)
AVF - arteriovenous fistula.		

## Further investigation

Recurrent secondary hyperparathyroidism was initially suspected due to the markedly elevated iPTH concentrations. Hyperplasia or increased activity of the autotransplanted parathyroid tissue was considered, and forearm ultrasound was contemplated to assess graft function. However, imaging was ultimately not pursued, as a repeated iPTH test using a sample drawn from the contralateral (non-grafted) arm yielded normalized results. This finding confirmed that the initial elevation was due to a sampling-site artifact rather than true graft hyperfunction, thereby resolving the diagnostic uncertainty without the need for further imaging.

## Solution

During routine iPTH surveillance in February 2025, a markedly elevated value (2869.4 ng/L) was reported from a sample collected in the right forearm. A second elevated result was observed in March, again from the same site. Despite the abnormal results, the patient exhibited no clinical signs or symptoms, and serum calcium and phosphorus were not significantly elevated. Upon chart review, it was noted that the parathyroid autograft had been placed in the right arm following TPTX-AT. A repeat iPTH sample was obtained from the left (non-grafted) arm, revealing a normalized value of 51.2 ng/L. This confirmed the spuriously high readings were due to localized secretion at the graft site.

## Discussion

Preanalytical variables are responsible for the majority of laboratory diagnostic errors, including issues such as mislabeling, improper sample collection, and inadequate transport or storage conditions (*7*, *8*). This highlights the need for careful attention to preanalytical factors, particularly the sampling site, in the interpretation of iPTH values. Our case adds to existing evidence by demonstrating that sampling-site errors can lead to spuriously elevated iPTH concentrations even months after parathyroidectomy. Notably, in our patient, falsely elevated iPTH concentrations were observed approximately five months after autotransplantation, suggesting that this preanalytical artifact may persist over the long term. This issue is particularly significant in dialysis settings, where routine laboratory testing may not consistently account for forearm graft sites. Persistent iPTH elevation due to localized secretion from the grafted arm highlights the importance of sampling from the non-grafted site and reinforces the need for long-term awareness of sampling locations to avoid diagnostic errors. Implementing standardized sampling protocols may prevent misinterpretation and reduce unnecessary diagnostic procedures.

## What YOU should / can do in your laboratory to prevent such errors

To minimize the risk of sampling-site–related errors in iPTH measurement, laboratories should ensure that the presence and location of parathyroid autografts are clearly documented within the Laboratory Information System (LIS). This allows staff to verify proper sampling sites during routine blood collection. Additionally, phlebotomy and nursing personnel should receive targeted training to avoid drawing samples from grafted forearms. Institutions are encouraged to establish standard operating procedures (SOPs) that define appropriate sampling practices for iPTH. When unexpectedly high iPTH concentrations are encountered, laboratories should consider retesting using a sample from the contralateral (non-grafted) arm to rule out localized secretion artifacts.

## Data Availability

No data was generated during this study, so data sharing statement is not applicable to this article.
